# A Composite Model of Wound Segmentation Based on Traditional Methods and Deep Neural Networks

**DOI:** 10.1155/2018/4149103

**Published:** 2018-05-31

**Authors:** Fangzhao Li, Changjian Wang, Xiaohui Liu, Yuxing Peng, Shiyao Jin

**Affiliations:** ^1^Science and Technology on Parallel and Distributed Laboratory, Changsha, China; ^2^College of Computer, National University of Defense Technology, Changsha, China

## Abstract

Wound segmentation plays an important supporting role in the wound observation and wound healing. Current methods of image segmentation include those based on traditional process of image and those based on deep neural networks. The traditional methods use the artificial image features to complete the task without large amounts of labeled data. Meanwhile, the methods based on deep neural networks can extract the image features effectively without the artificial design, but lots of training data are required. Combined with the advantages of them, this paper presents a composite model of wound segmentation. The model uses the skin with wound detection algorithm we designed in the paper to highlight image features. Then, the preprocessed images are segmented by deep neural networks. And semantic corrections are applied to the segmentation results at last. The model shows a good performance in our experiment.

## 1. Introduction

Wound observation and analysis are the basis of the wound treatment. The process of them includes area measurement and tissue analysis. The manual methods require lots of time and labor. Moreover, the results are inaccurate and the processes are unhygienic. Wound segmentation is the basis of automatical wound area measurement and tissue analysis which can avoid these problems of the manual methods. While saving manpower and material resources, it also makes it easier to establish electronic medical record for the supplementary medical care.

Wound segmentation can be achieved by using the methods based on the traditional algorithms and deep learning. Traditional methods [1–3] need to manually design wound features for the classification algorithms used in the wound image processing, such as wound segmentation, tissue detection, and wound diagnosis. Deep learning [4–7] is popular with recent years. It takes advantage of the powerful feature extraction ability of the trained deep neural networks to process images. Deep neural networks (DNN) have excellent performances in image classification, recognition, and segmentation. However, due to the lack of labeled wound images, the researches on wound segmentation based on deep learning [5] are insufficiency.

Traditional methods are limited to artificial description capabilities, while deep learning methods are limited to training data. This paper presents a composite model that combines the traditional methods with deep neural networks. We use our semiautomatic annotation software to create a labeled wound database. Based on the database, the model uses skin with wound detection algorithm designed in this paper to highlight image features and segments the preprocessed images by DNN. The results are corrected semantically by applying the traditional methods.

Our work mainly includes the following innovations:

(1)  A composite wound segmentation model combining traditional methods with the deep learning is proposed.

(2)  In combination with watershed and dynamic threshold algorithms, we propose a method of kin with wound detection.

(3)  Based on the research of different preprocessed train-test groups, we get an optimal train-test group.

## 2. Related Works

Many traditional methods select an appropriate color space for wound segmentation firstly. Then, the color and texture features of the wound image are extracted to classify each pixel or pixel block by applying the classification algorithms. Mukherjee et al. [8] proposed a traditional wound segmentation framework. They preprocessed the data by filtering the images and applied the fuzzy divergence algorithm to the S channel of the images in the HIS color space for wound segmentation. Veredas et al. [2] proposed a hybrid approach based on neural networks and Bayesian classification for tissue classification. In their paper, the mean shift algorithm was used to smooth the raw images, and the algorithm of regional growth was applied to segment the images. Dhane et al. [9] constructed a similarity matrix. The elements of the matrix are the gray-level fuzzy similarity values combined with the image space information. They chose the Db channel from 26 color channels by calculating the color contrast and used the fuzzy spectral clustering method to segment the wounds based on the similarity matrix. Their results were corrected by the morphological operation.

**Figure 1:**
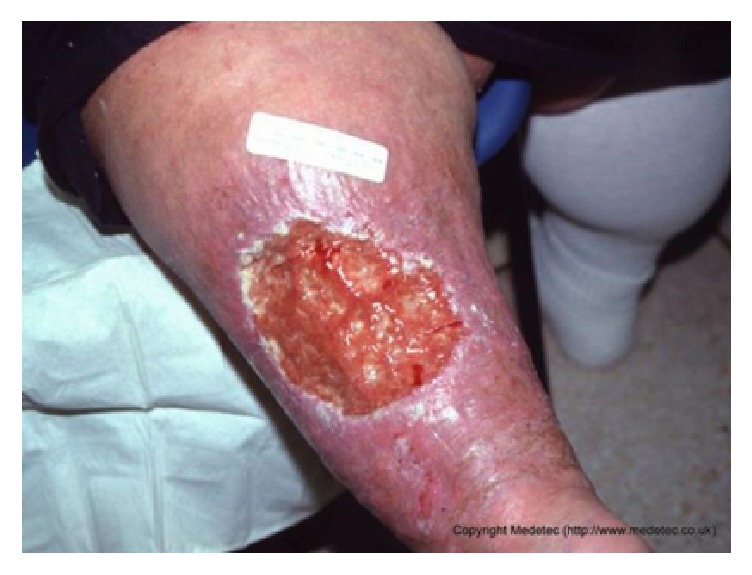
One of our images. Compared with the images shown in paper [10], the backgrounds of our images are more complex.

The wound images used in the above papers generally have few environmental backgrounds, or their environmental backgrounds are simple. As shown in Figure 1, our image has a complicated background.

Due to the lack of labeled wound images, there are few studies on the use of deep learning methods for wound segmentation currently. Wang et al. [5] used 9-layer networks, and their training set consists of 500 wound images. The depth of the neural networks used in this paper is not deep enough, and the training data is small, so, their improvement in the result accuracy is not significant. At present, there are many methods based on deep learning for image segmentation. The structure of Fully Convolutional Networks (FCN) [4] used in our research is a typical neural networks structure for image segmentation. FCN replaces the fully connected layers in the classification networks with convolutional layers, which makes the networks more suitable for image segmentation. The key part of the deep neural networks is used to extract the image features. This part has many different structures in the classic networks, such as VGG [6], and resnet [7]. Based on the analysis of our task, we choose the MobileNet [11] to extract the image features. MobileNet can adjust the complexity of the networks conveniently by setting two hyperparameters.

In order to exploit the advantages of the traditional methods and the deep learning, we propose a composite wound segmentation framework that combines these two approaches. In the application of traditional methods, this paper presents an algorithm of skin with wound detection. Dividing a wound from the skin is easier than dividing it from the image with a complex background. So, we segment the skin with wound from the image first. Currently, for the study of skin detection, many articles [13–15] convert images to the YCbCr color space to complete the task. That is because the distribution of the skin color in this space is more concentrated. Hsu et al. [13] used a parametric elliptic model based on the YCbCr color space to detect the skin. Their experiments show that, in the CbCr subspace, pixels of healthy skin fall roughly in an oval area. As health skin color distribution is more uniform, their method can effectively detect the healthy skin. However, the color distribution of the wound is complex and does not satisfy the above ellipse model. Therefore, we cannot directly use this method to detect injured skin. Kim et al. [16] applied the latest deep learning for skin detection. These methods have good performance in skin detection. But, the datasets ECU [17] and Pratheepan [18] used were both labeled data onto healthy skin and we did not find any labeled data onto injured skin. So, these algorithms cannot be used directly.

## 3. Work Flow

We collect wound images from the Internet (http://www.medetec.co.uk/files/medetec-image-databases.html. http://www.handsurgery.cn) and hospital to build our database for training deep neural networks. However, it is hard to improve the accuracy of wound segmentation by adding a large amount of training data at present, since manual labeling for wound images requires lots of time and labors.

**Figure 2:**
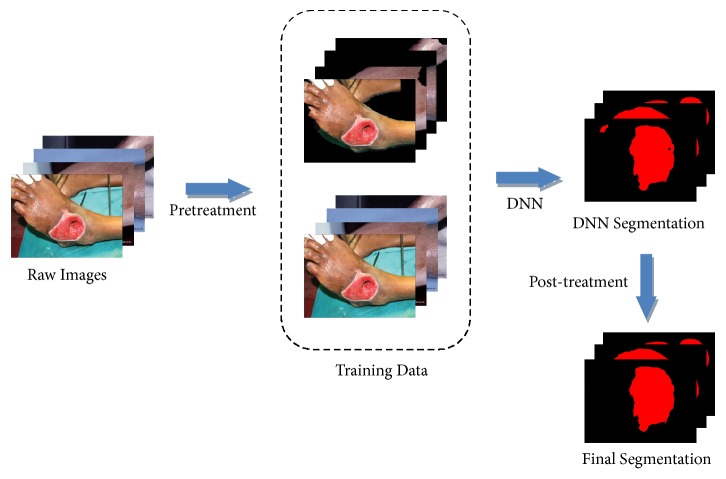
The architecture of our composite model. Raw images are preprocessed by skin with wound detection algorithm to remove the environmental backgrounds. Then, the training data composed of the preprocessed images and the raw images are normalized, cropped, and deformed. The DNN is trained to segment the testing data. At last, the segmented results are corrected semantically.

In this paper, our task is to segment the wound from the image. It is the basis of the wound tissue analysis, and it can be used to measure the area of the wound directly. This task can simply be considered as a problem of the two-class classification. We use the algorithm of skin with wound detection to remove the environmental background from the training and testing images. The preprocessing could simplify the segmentation task and expand the training set. At the same time, we use the traditional methods of image processing to semantically correct the results of the deep neural networks. The workflow of our model is shown in Figure 2.

The first step is the preprocessing of wound images. Its goal is to highlight the wound features and augment the data. This process includes the removal of the environmental background of the wound image and the preprocessing of the training data. The background removal is to simplify the segmentation task and to highlight the wound features. Training preprocessing can improve the quality of networks training and feature extraction.

The second step is the preliminary wound segmentation by using deep neural networks. Based on the preprocessed images, we set the structure of the deep neural networks reasonably and use it to complete the preliminary segmentation of the wound images. We select the best networks segmentation training mode by testing different train-test groups.

The last step is the semantic correction of the results. According to the semantic features of the wound images, the segmented images are semantically corrected. Semantic features of the wound images include the relationship between the wound and the skin such as surrounded and adjacent, the wound area without holes, and the ratio between the wounds.

## 4. Work Details

In our model, we use the traditional methods to remove the backgrounds of the images and semantically refine the segmentation results. DNNs are used to extract and mine the image features for wound segmentation.

### 4.1. Skin with Wound Detection and the Removal of the Background

**Figure 3:**
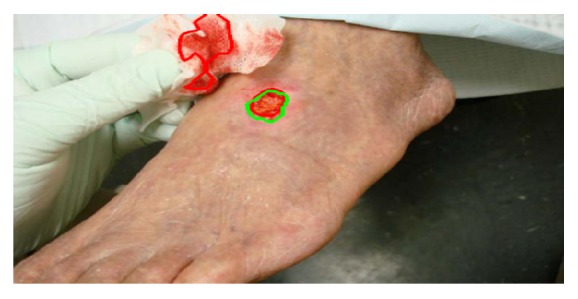
The green line indicates the wound, the red line indicates a similar background, and such a background can be easily misjudged as a wound. If we delete it before segmentation, we will simplify the task.

A complex background may contain regions that are similar to the target. In the task of image segmentation, it is difficult to distinguish a similar background region from the target. In images, features that measure similarities can be color, texture, or location. Our images contain lots of background regions similar to the wounds. In our task, due to the cost of manual labeling, it is difficult to improve the segmentation accuracy of DNN by adding training data. Therefore, we perform skin with wound detection and environmental background removal on the wound images to simplify the task, so as to improve the accuracy of wound segmentation without increasing the complexity of DNNs or adding training data. As shown in Figure 3, a nonskin background region is similar to the wound and can be easily misidentified. If we remove the nonskin background before the segmentation, we can simplify the task and improve the accuracy.

Applying skin with wound detection to wound images is to remove their environmental backgrounds, so as to highlight the target features of the wound images. In this paper, we need to apply the algorithm of the background removal twice. The first background removal is applied before DNN segmentation; it requires a complete retention of the wound. The second background removal is applied to the results of DNN, and it requires removing the background as much as possible. We propose a skin with wound detection algorithm that can achieve the two goals by changing some threshold parameters.

**Figure 4:**
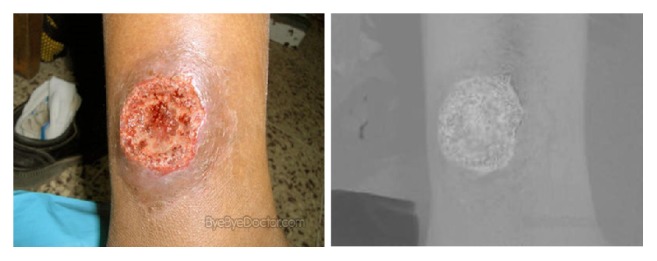
RGB color space of an image and its Cr channel in the YCbCr color space.

Studies have shown that the distribution of the Cr values of skin pixels is concentrated[13]. As shown in Figure 4, there is a clear difference between the skin and the background in the Cr channel. We complete the skin detection based on this character of skin pixels.

**Figure 5:**
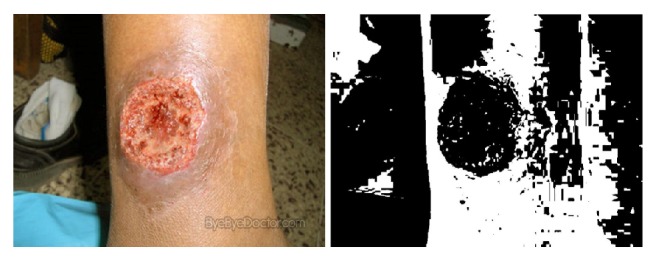
Skin detection with fixed threshold of Cr channel in YCbCr color space.

Through the statistical analysis of the skin pixels in our data, we found that most of the Cr values of skin pixels ranged from 135 to 160. However, the backgrounds of the images to be processed are complex, and the colors of the images vary widely. Moreover, we also need to consider the wounds with different colors. Therefore, if we simply set the threshold for skin detection to the above values, the algorithm will be lack of robustness. As shown in Figure 5, a fixed threshold of skin detection will misjudge lots of healthy skin pixels and most of the wound pixels.

Our approach is based on two assumptions. (1) In an image, the skin pixels are similar in color. (2) Proportion of skin and wound in an image will not be too small (no less than 7%). The images with small proportion of skin and wound are difficult to be used for the observation and analysis of the wound; that is, they have little application value. Moreover, such images are rare and they have little effect on the experimental results. We usually delete such images when creating the database.

**Figure 6:**
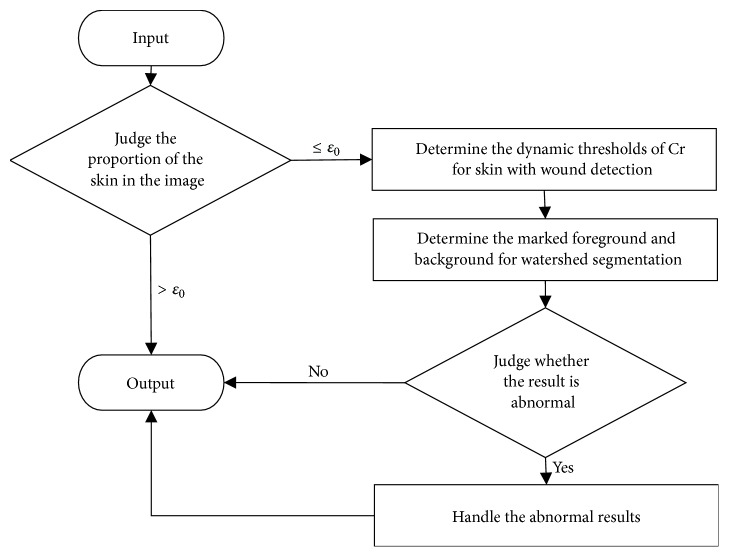
Algorithm flow of skin with wound detection.

Based on the above assumptions, as shown in Figure 6, our method is mainly divided into the following steps.

**Figure 7:**
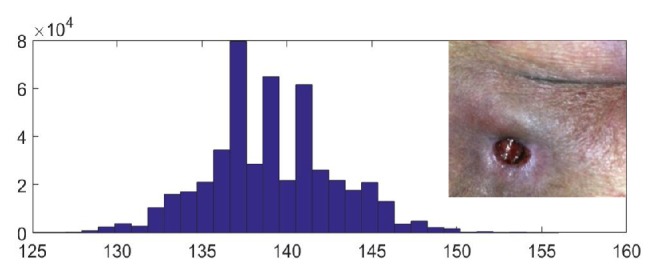
Cr histogram of an image without complex backgrounds, the top right corner is the image.

The first step is to predict the proportion of the skin area in the image. If the proportion is large, we output the image directly. As shown in Figure 7, the Cr values of pixels in the image are mostly concentrated in the interval [130, 150]. The width of the interval is 20. In order to increase the robustness of the algorithm and based on the statistical interval [135, 160] of skin pixels, we assume that if the proportion of pixels whose Cr values in some interval with width d=25 exceeds a certain value *ε*, then these pixels are all considered as skin and the environmental background in this image can be neglected. The judgment formula as shown in formula (1).

(1) p=NLN>ε,L:pixel ∣ Crpixel∈μ0+δ,μ0+d+δ

where p is the predicted proportion of the skin in the image, N_L_ is the number of pixels with Cr values in an interval with width d, and these pixels are predicted as skin pixels. N is the total number of pixels of the entire image. *ε* is the judgment threshold. The value of *ε* is set to 93% at first and then fine-tuned to 92% in our experiment. In an image, the pixel set with a very concentrated distribution of Cr values will not be a mixture of the skin and the background. If these pixels are the pixels of the background, then the proportion of the skin will be less than 7%; it does not meet our second hypothesis. So we predict that such pixels are the pixels of the skin. *μ*_0_ is the initial value of Cr, which we set as the minimum Cr value of pixels in each image. *δ* is the adjustment value used to slide the interval [*μ*_0_, *μ*_0_ + d].

The second step is to set the dynamic thresholds of Cr for skin with wound detection. For images with small predicted proportion of skin, we need to determine the Cr thresholds for pixels of the skin with wound. We want to find an interval with a bigger proportion of pixels and an appropriate width. The operation is as follows. (1) A set S_m_ is constructed by finding the minimum points in the Cr histogram of the image and removing the minimum points at both ends. S_m_ = {(Cr_i_, H_i_) | H_i_ ∈ min⁡(H)}, where H is a set of pixel statistics for each Cr value of the pixel in the image. (2) We set two initial Cr intervals A = [*μ*_L1_, *μ*_L2_] and B = [*μ*_H1_, *μ*_H2_]. Then, the minimum points Cr_A_ and Cr_B_ in S_m_ that are in the A and B intervals are located. Then, the distance L_m_ between Cr_A_ and Cr_B_ and the proportion p_m_ of pixels with Cr values in interval [Cr_A_, Cr_B_] in the image are calculated. (3) We judge L_m_ and p_m_. If L_m_ ∈ [L_min_, L_max_] and p_m_ > *ε*_0_ (*ε*_0_ = L_m_/L in this paper, L is the Cr distribution interval width of 95% pixels in the image), Cr_A_ and Cr_B_ are the upper and lower thresholds we need. Otherwise, we translate A and B and repeat steps (2) and (3) until two thresholds that satisfy the condition are found. Based on the width of the statistical interval [135, 160], we set *μ*_L1_ = 130, *μ*_L2_ = 140, *μ*_H1_ = 155, *μ*_H2_ = 165, L_min_ = 15, and L_max_ = 30 in the paper. The algorithm pseudo code is shown in Algorithm 1.

**Figure 8:**
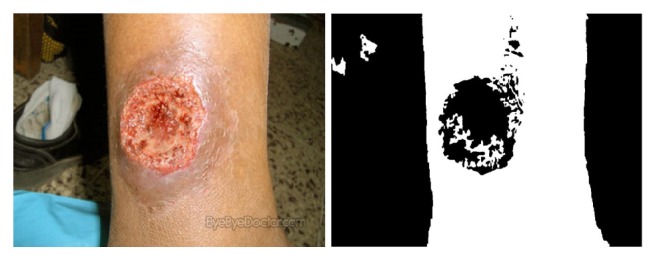
Skin detection with dynamic threshold of Cr channel in YCbCr color space.

Our experiments show that the thresholds determined by the above method can better preserve the wound area and have better robustness, as shown in Figure 8. However, this method usually cannot preserve the complete wound area and still needs further process.

**Figure 9:**
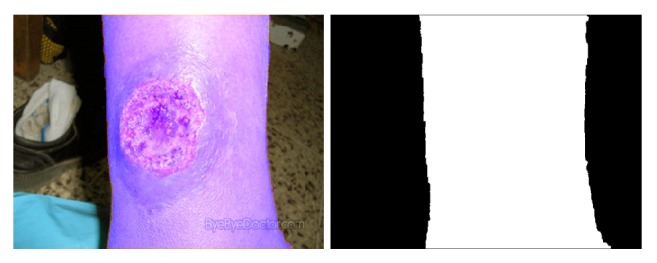
Result of the skin with wound detection.

The third step is to use the marker-based watershed method to the further skin with wound segmentation. In the marking process, we modify the area of predicted skin in the second step, such as filling holes and morphological erosion. The resulting area is marked as foreground. The morphological erosion here uses circular structure element with diameter d_1_ = 10. At the same time, the marked background is obtained from the morphological erosion of the previous background region. And the diameter of the structure element is d_2_ = 20. Each of our images sizes is larger than 512×512. Using such structural elements will not lead to the vanishing of the foreground if the injured skin area is small. This process can eliminate some small areas that are misidentified as foreground. Obviously, skin detection is binary segmentation. However, more than two regions may be obtained by this marking method, which will lead to more unstable postprocessing. Here, we connect the regions of the foreground and the background severally. For multiple foreground regions, we connect their centers of gravity with thin lines to create a new marker region. We cannot handle the background area by this way; otherwise the foreground and the background may be connected. So, we connect the background areas outside the image. This method is to add a circle border around the Cr graph, and the value of the border elements is the mean of the predicted background. This border is marked as the background; it can connect all the background areas. Finally, the watershed algorithm is applied to the marked gradient map to detect the skin area. The result is shown in Figure 9.

**Figure 10:**
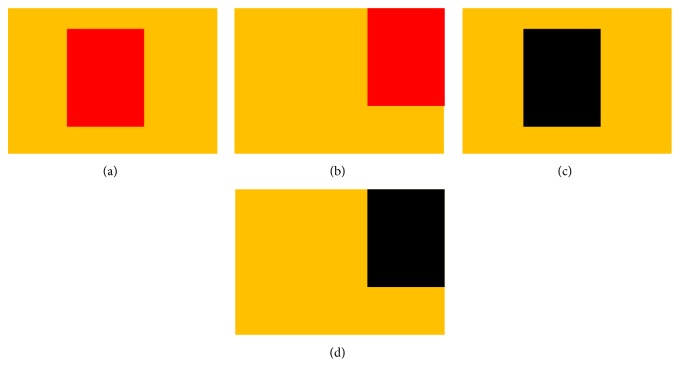
The relationship between the skin and the wound.

The last step is the handling of anomalies. Due to the complexities of our data backgrounds and wound compositions, there are still some anomalies in the above segmentation results. As shown in Figure 10, the relationship between the skin and the wound can be divided into the following categories.

The first category shows in, Figure 10(a), that their color difference is small; the wound is surrounded by healthy skin. The second category shows, in Figure 10(b), that their color difference is small; the wound is not surrounded by healthy skin. The third category shows, in Figure 10(c), that their color difference is big; the wound is surrounded by healthy skin. The fourth category shows, in Figure 10(d), difference their color difference is big; the wound is not surrounded by healthy skin.

Our method fills the holes in the foreground areas, so, for the first three images, all the wounds are included in the predicted skin. However, for the fourth images, parts of the wound may be missed. Our experiments show that the color of the missing wound is close to black.

**Figure 11:**
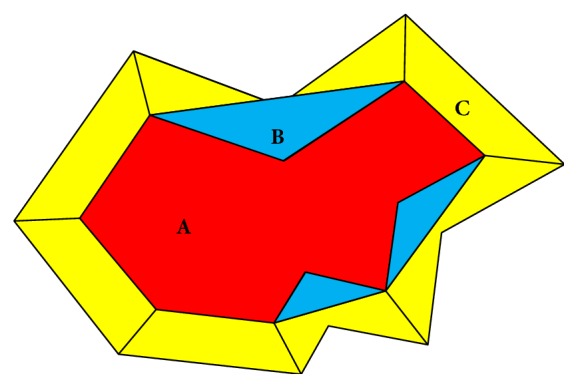
Schematic of the relabeled regions in the skin with wound detection.

For this problem, we cannot predict the relationship between the skin and the wound in an image. Then, we choose to solve the problem by applying the watershed algorithm twice after getting the predicted skin with wound areas. At first, we find the morphological dilation area of the skin with wound and its convex bounding boxes. The corresponding vertices between the two convex bounding boxes are connected by solid lines. Then we find small areas that need to be further marked. As shown in Figure 11, the red area A represents the injured skin, the blue area B represents the difference between A and its convex bounding box, and the yellow area C represents the difference between the morphological dilation area of the predicted skin and the convex bounding box. Regions separated by black solid line in B and C are the small regions that we need to make further judgments. Next, for these small regions, we observe their RGB values. If the color of a small area is not close to black, the region is deleted; if the color of a small area is close to black, the sum of RGB variances of the region is calculated; in the case of a background, this value is usually low and such a region is deleted; otherwise, we keep this region as a foreground area. Finally, the newly obtained foreground areas are connected with the marked area in the third step, and a new marked area is obtained. Once again, the watershed algorithm is used to obtain the result of skin with wound detection. In this way, we will not miss the wounds in the fourth images.

### 4.2. Training Data Preprocessing and Wound Segmentation Based on Deep Learning

**Figure 12:**
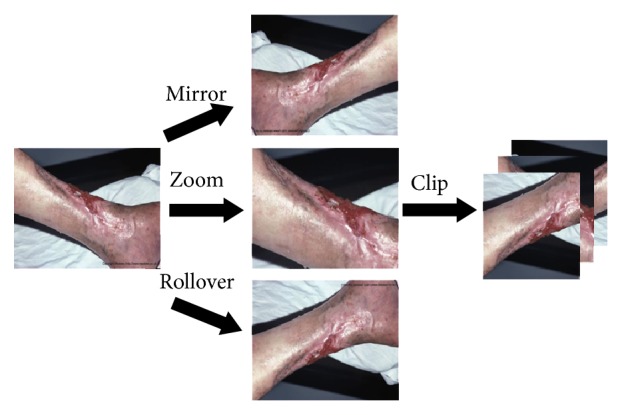
Image deformation and cropping. Left is the raw image; the deformed images are showed in middle. Right is the clipping results of deformed images.

We add the background-removed images to the training set and then preprocess the images in training set. The process includes image standardization, image deformation, and cropping.

(*1) Image Standardization*. This process is mainly to normalize the means and variances of the images. We use (2) to process the input images.

(2) $$\begin{array}{c} X_{N}=\frac{X-\mu_{X}}{\sigma_{X}} \end{array}$$

where X is the input data, *μ*_X_ is the mean of X, and *σ*_X_ is the variance of X. The standardization of the image can avoid the impact of some unrelated factors and highlight the image features, so as to avoid the training falling into some local optimums.

(*2) Image Deformation and Cropping*. In order to further expand the training set and enhance the robustness of the networks, we deform and crop the images in training set. As shown in Figure 12, this process can improve the generalization of the networks, especially when the training data set is small. In this paper, the input image size of the networks is 512 × 512. In our experiment, one raw image can be converted to 60 training images.

The excellent DNN with a large number of parameters require huge amount of training data. Due to the lack of the training data, we need to reduce the parameters. So, we take the MobileNet as the main structure; then, we use two interpolation layers to replace its fully connected layers. The output layer is a binary fully connected layer in our DNN. These operations greatly reduce the number of parameters without affecting the accuracy of the networks.

**Figure 13:**
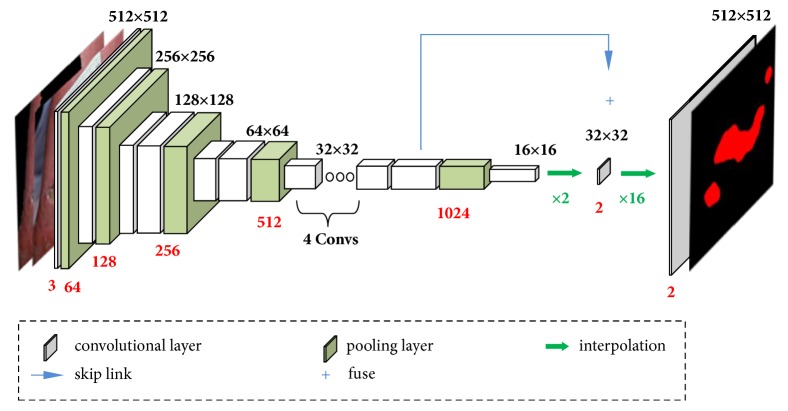
Schematic of our deep neural networks framework. In the figure, the white box represents the convolutional layers, the green arrow represents the upsampling, and the blue arrow represents the fusion of the data. The red number below each layer represents the number of output feature channels.

Our networks structure is shown in Figure 13. The backbone of the networks is 13 convolution layers of MobileNet (a pair of depthwise and pointwise layers is considered as one layer). We upsample the output of the last layer of convolutional layer and fuse the output of the previous convolutional layer before pooling to ease the problem of loss of location information. Finally, the fusion result is upsampled sixteen times to ensure that the output and input have the same resolution. The activation function is used to determine if each pixel belongs to the foreground. We experimentally find that a network structure that fuses one convolutional layer has the best segmentation accuracy.

The numbers of parameters in our models are shown in Table 1.

### 4.3. Semantic Correction

After wound segmentation by DNN, we correct the results further based on the semantic analysis of the wound images. The input of the semantic correction is the output of DNN. Wound semantic correction mainly includes the following contents.

(*1) Fill the Holes*. We assume that the wounds do not enclose the healthy skin, which means that there should be no holes in the predicted wounds. Based on this assumption, we use morphological processing [19] to fill the holes in the wounds.

(*2) Remove the Minor Noise*. Due to the image noise and uneven exposure, the segmentation results will contain some minor errors. All of our wounds are judged, if the wounds that are too small or relatively small will be deleted. See formula (3) for the method of judgment.

(3) $$\begin{array}{c} A_{del}<\varepsilon_{d}=\min\left(\;\max\left(\;A\;\right)\times P_{d},A_{d}\;\right) \end{array}$$

where A is the area set of all the wounds in the image to be corrected. P_d_ is the minimum area ratio of other wounds to the largest wound in A (P_d_ = 5% in this paper) and A_d_ is the smallest reserved wound area (in this paper, A_d_ = 500).

(*3) Wound Correction Based on the Skin Detection*. Segmented wound areas should be the subset of the predicted skin area. Although most of the background has been removed from the preprocessing, in order to preserve the complete wounds, the above predicted skin areas will contain some backgrounds. These backgrounds are similar to skin and wounds. Therefore, in the results of wound segmentation, there are some errors of identifying the background as wound. Next, we will change some of the thresholds in the skin with wound detection to delete more backgrounds. The parameters to be adjusted are d_1_ and d_2_. Here, d_1_ = 20 and d_2_ = 10. However, this operation may result in missing parts of the wounds. So, in the process of this step, we make a judgment according to formula (4) for each predicted wound.

(4) $$\begin{array}{c} \frac{A\left(R_{s}\cap R_{w}\right)}{A\left(R_{w}\right)}>\varepsilon_{s} \end{array}$$

where R_s_ is the skin region predicted in the postprocessing and R_w_ is the wound region to be corrected. The numerator is the area of the overlap between the two regions and the denominator is the area of the wound region. If this ratio is greater than the threshold *ε*_s_ (*ε*_s_ = 0.75 in this paper), this wound region will be preserved; otherwise, this wound region will be deleted.

Compared with conditional random field(CRF), our correction method is based on the general semantic features of wound and is more robust. At the same time, our algorithm runs faster than pixel-based CRF.

## 5. Experimental Results and Analysis

We implement the model in the paper and test it on our database. Images in our database are collected and labeled by ourselves.

### 5.1. Database Source

**Figure 14:**
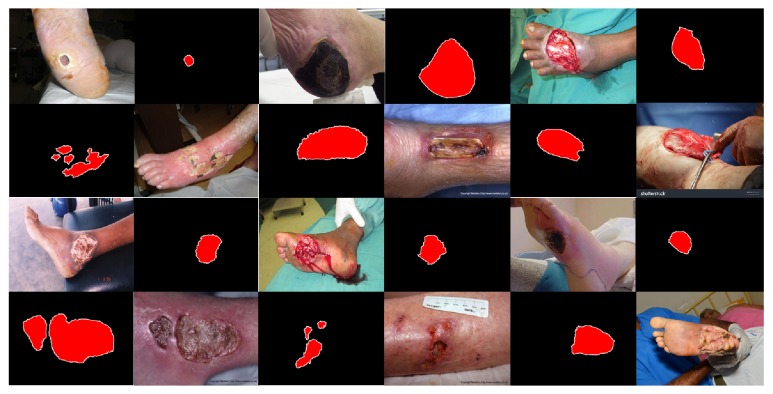
Wound images and labeled images in our data.

**Figure 15:**
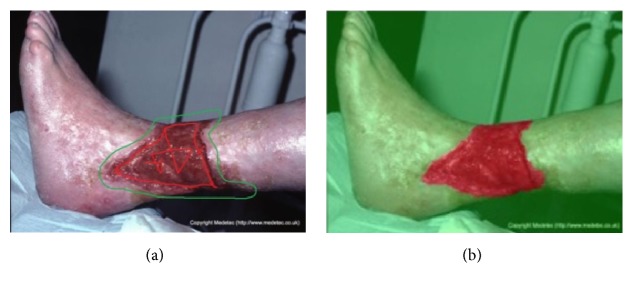
(a) The schematic of the foreground and background marking. The red curve is the foreground marker, and the green curve is the background marker. (b) Results of applying the watershed algorithm based on the marked gradient map of the image. Red region is the wound. The interactive interface of the software is shown in (a). The labeling staff performs rough marking on this interface, and the marked curve can also be corrected repeatedly.

We sorted out 950 wound images from the Internet and the hospital. Among them, 389 images were captured by clinicians in the hospital and the rest are from the Internet. The size of those images includes 800 × 600, 700 × 700, 600 × 800. Lighting environment of these images is indoors with artificial light, and the camera enables auto flash function. In these images, most of the wounds are located on the human lower extremities and the others (about 20 images) are located on the abdomen, the upper extremities, or the head. Most of the wounds are in the center of the images; about 30% of the images also have wounds at the edges and corners, but these images contain complete wounds. The type of wounds includes wound caused by diabetic foot complications, pressure ulcers, and burns. On some wound surfaces, there are drugs for treatment. Guided by the medical staff, we labeled these images by using our semiinteractive wound labeling software. Parts of the labeled images are shown in Figure 14, and the process of our software is shown in Figure 15. Because the segmentation edges are determined by the combination of the experience of the medical staff and the watershed algorithm, the annotation is authoritative and normative.

### 5.2. Training and Testing of DNN

We use the computing platform with 3.4GHz, 16GBRAM, and NVIDIA GeForce GTX1070 configuration to complete the experiment.

In the process of training DNN, we use batch normalization [20] to enhance the robustness of the networks. The regularization technique we used is weight decay, and the optimisation algorithm we used is Adam. We set the learning rate lr = 0.01 × (0.5)^max⁡(0, (step/5000−2))^. The size of the minibatches is 5. In order to test the generalization ability of the networks, we use the method of hold-out to test our DNN. The 950 images are divided into two groups, of which 190 belong to the test set and 760 belong to the training set. The sequences of images in these sets were randomly generated. We test our DNN five times and the averages of these test results are displayed in the paper. The training is stopped at 50000 steps. One step is the process of dealing with a minibatch, so the number of epochs is 50000/(760/5)=329. We use tensorflow to design and test our networks and speed up the training by using 1 GPU which is NVIDIA GeForce GTX1070. The training phase is 8 hours.

### 5.3. Image Segmentation Evaluation Index

Image segmentation evaluation index includes sensitivity, precision, intersection over union(IoU), and dice similarity coefficient(DSC). We choose the precision and the IoU to analyze our model. They are defined as

(5) Precision=NTPNTP+NFP

(6) IoU=NTPNTP+NFP+NFN

where N_TP_ presents the number of the true positive, N_FP_ is the number of the false positive, and N_FN_ is the number of the false negative.

In fact, the IoU is more in line with our application because it takes into account both false positives and false negatives.

### 5.4. Model Results Analysis

**Figure 16:**
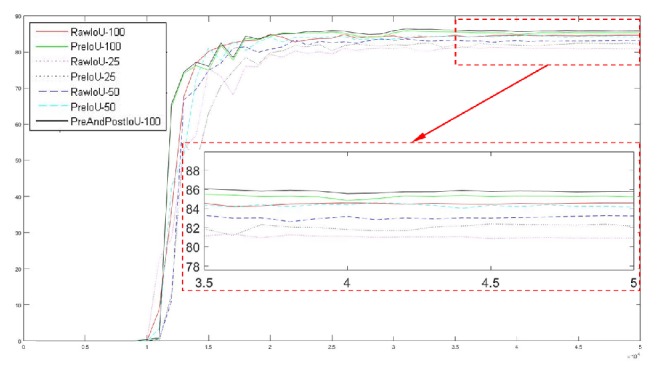
Test results for different models. The abscissa is the number of steps in training and the ordinate is the IoU obtained by testing the test data. One step is the process of dealing with a minibatch. RawIoU-100 represents the IoU of the original networks with DM = 100%. PreIoU-100 represents the IoU of the pretreatment model with DM = 100%. PreAndPostIoU-100 represents the IoU of the complete model with DM = 100%.

In order to test the validity and robustness of our model in networks with different sizes, we change the size of the DNN by changing the depth multiplier (DM) of MobileNet. As shown in Figure 16, we test the performance of our model in networks with DM equal to 25%, 50%, and 100%. The original networks refers to a model that uses only the deep learning to segment the wounds. The preprocessing model refers to the model that adds the preprocessed images to the input of the original networks. The preprocessed images are the images that have their backgrounds removed by skin with wound detection. Compared with the original networks, the IoU of the preprocessing model has about 1% improvement. Our complete model is a model that adds the postprocessing of the semantic correction to the preprocessing model. We have tested the complete model with DM = 100% and its IoU is 1.5% better than the original networks. In addition, we find that the IoU of the preprocessing model with DM = 50% is close to the IoU of the original networks with DM = 100%. This shows that our method can effectively reduce the parameters of the networks with the same precision.

Table 2 shows the test results numerically. In the table, mIoU refers to the average of the stationary part of the IoU curve and MaxIoU is the maximum value of the IoU curve. Raw represents the original networks. Preprocess represents the preprocessing model and PreAndPost represents the complete model. 0.25, 0.5, 1.0, etc. denote the value of DM.

From Table 2, we find that the IoU-related indicators of our complete model are the best, but its precision is not improved compared to the preprocessing model. This shows that our postprocessing mainly corrects the target areas that are predicted as the backgrounds.

### 5.5. Analysis of Train-Test Group

In order to find a train-test group with the best segmentation results, we test six groups of these two sets. These groups include the following:

First Group: Training set is composed of the raw data. Test set is composed of the raw data.

Second Group: Training set is composed of the raw data. Test set is composed of the preprocessed data.

Third Group: Training set is composed of the raw data and the preprocessed data. Test set is composed of the raw data.

Fourth Group: Training set is composed of the raw data and the preprocessed data. Test set is composed of the preprocessed data.

Fifth Group: Training set is composed of the preprocessed data. Test set is composed of the preprocessed data.

Sixth Group: Training set is composed of the raw data and the preprocessed data. Test set is composed of the raw data and the preprocessed data.

The test results are shown in Table 3. We can find that the results of the fourth group are the best.

As shown in Table 3, images in the first group are all unprocessed. Images in the test set of the second group are replaced with the preprocessed images. Compared with the first group, the segmentation accuracy of the second group has a slight improvement. This is because our preprocesses can remove some of the background similar to the wound and avoid some errors when testing the segmentation. If images in training set are replaced by preprocessed images, which is the fifth group, the segmentation accuracy is further improved. This means that our preprocesses can simplify the task of wound segmentation. When images in training set and test set have been applied the same appropriate preprocessing, the task can be simplified and the accuracy can be improved. The fourth group adds the unprocessed images to the training set of the fifth group. Increasing the training data reasonably can enhance the generalization ability of the networks and improve the segmentation accuracy. However, the training set of the third group has added the preprocessed images to the training set of the first group, which in turn reduced the segmentation accuracy. Since the test set is not preprocessed, adding the preprocessed image to the training set does not add extra valid information for the test set but increases some interference and reduces the segmentation accuracy. The test accuracy of the sixth group is the mean of the third group and the fourth group.

### 5.6. Comparison of Existing Methods and Results Shown

Some of the existing methods and their segmentation results are shown in Table 4. We implemented the method of Wang et al. [5] in conjunction with the tricks used in this paper and tested it with our database. Our method works best on all evaluation index.

**Figure 17:**
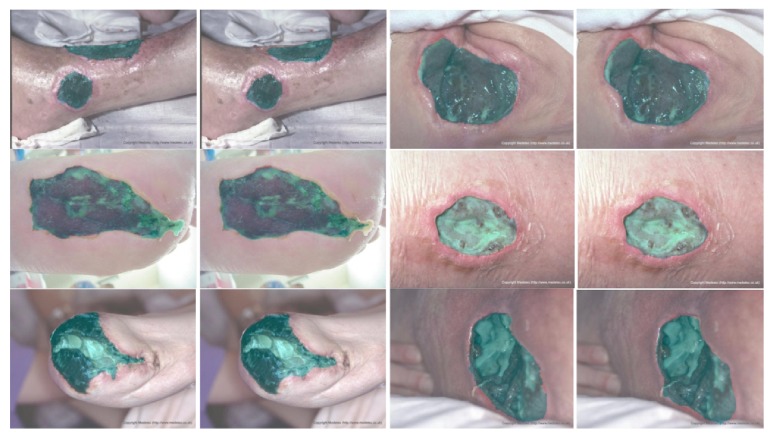
Results show. Left is ground truth and right is the results of our model.

Finally, we show some of our results in Figure 17. The green areas are labeled as the wound areas. In the figure, ground truth is on the left and our results are on the right.

### 5.7. Limitations of Our Model

In our model, some limitations need to be researched further. First, there are still some nonskin backgrounds that are misjudged to be skin in the results of skin with wound detection. This problem leads to some nonskin backgrounds being misjudged as wounds in the final results. Our skin with wound detection algorithm needs further improvement. Second, due to the fact that a 16-fold upsampling is used in our neural networks, the results in our segmentation results are too smooth compared with the ground truth, as shown in Figure 17. We will use the CRF algorithm in future studies to further refine our results. Third, our traditional algorithm has a high dependency on the statistics of the database. The parameter values (thresholds, histogram interval ranges, etc.) have been determined on the basis of the latent statistics of our datasets. And, all the images in our dataset should be bigger than 521 × 512 pixels. Finally, our model is more complex than previous methods; it consists of three processes, preprocessing, DNN classification, and postprocessing. Our model cannot achieve end-to-end training due to its complexity.

## 6. Conclusion

We propose a framework for wound image segmentation that combines traditional digital image processing methods with deep learning methods. The traditional methods are mainly used for the semantic processing and correction of the data in DNN. With regard to the traditional method, we propose a skin with wound detection algorithm that has good robustness and plays a very good role in our model. Through experiments, we found a train-test group with the best segmentation results.

## Figures and Tables

**Algorithm 1:**
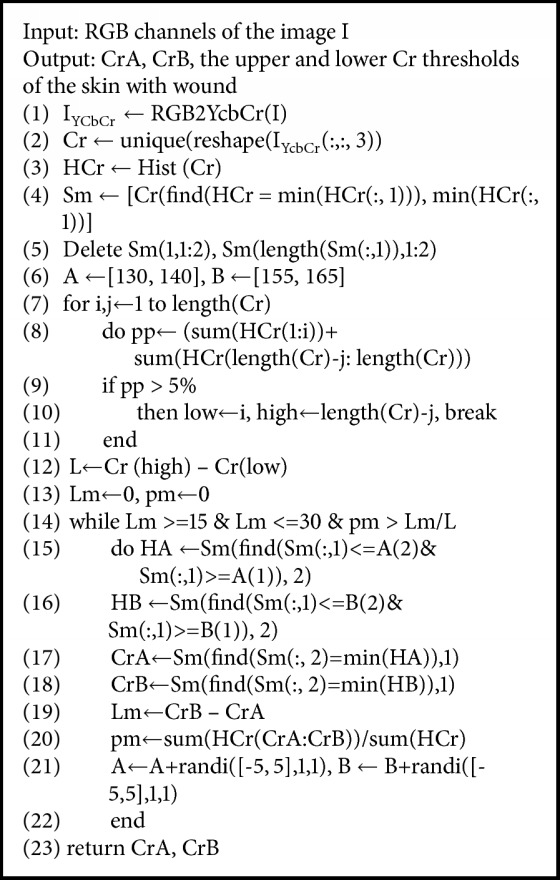
Determine the dynamic thresholds.

**Table 1:** Numbers of parameters in our models.

Width Multiplier	Million parameters
1.0 Model	3.19

0.5 Model	0.79

0.25 Model	0.20

**Table 2:** Results of different models.

Model	mIoU (%)	MaxIoU(%)	Precision (%)
Preprocess-1.0	85.3262	85.8030	**94.9430**

Raw-1.0	84.4080	84.8738	93.5520

Preprocess-0.5	84.2682	84.6251	94.2579

Raw-0.5	83.0970	83.5808	92.3514

Preprocess-0.25	82.0241	82.4051	92.9335

Raw-0.25	80.9700	81.3167	90.8778

**PreAndPost-1.0**	**85.8823**	**86.4018**	94.6906

**Table 3:** Test results of different train-test groups.

Train-Test	mIoU (%)	MaxIoU (%)	Precision (%)
First group	84.4080	84.8738	93.5520

Second group	84.6801	85.2562	94.7440

Third group	83.8502	84.7383	93.1100

Fourth group	**85.3262**	**85.8030**	**94.9430**

Fifth group	85.2744	85.6295	93.9758

Sixth group	84.7999	85.2972	93.7257

**Table 4:** Comparison of existing methods. The numbers in parentheses are the numbers of images in the test sets.

Related work	Number of images	Approaches	Degree of complexity of the final solution	Precision(%)	mIoU(%)
Yadav et al. [3]	77(77)	Fuzzy c-means	simple	86.78	-

Dhane et al. [12]	105(105)	spectral clustering	simple	91.80	-

Dhane et al. [9]	70(70)	fuzzy spectral clustering	simple	87.30	79.0

Wang et al. [5]	650(150)	deep CNN	middle	-	47.30

Wang et al. [5]	950(190)	deep CNN	middle	93.43	73.36

**Ours**	**950(190)**	**deep CNN+**	**complex**	**94.6906**	**85.8823**

## Data Availability

The data of labeled wound images used to support the findings of this study have not been made available because the data is still in the process of expansion and collation. Moreover, the data are obtained through cooperation between us and the hospital. It must be approved by both parties before it can be disclosed.
