# Corrigendum to “A Composite Model of Wound Segmentation Based on Traditional Methods and Deep Neural Networks”

**DOI:** 10.1155/2018/4967290

**Published:** 2018-09-12

**Authors:** Fangzhao Li, Changjian Wang, Xiaohui Liu, Yuxing Peng, Shiyao Jin

**Affiliations:** ^1^Science and Technology on Parallel and Distributed Laboratory, National University of Defense Technology, Changsha, China; ^2^College of Computer, National University of Defense Technology, Changsha, China

In the article titled “A Composite Model of Wound Segmentation Based on Traditional Methods and Deep Neural Networks” [1], the first affiliation was incomplete. The revised affiliations' list is shown above.
